# [Retracted] Radiation-inducible silencing of uPA and uPAR *in vitro* and *in vivo* in meningioma

**DOI:** 10.3892/ijo.2026.5890

**Published:** 2026-05-11

**Authors:** Venkateswara Rao Gogineni, Arun Kumar Nalla, Reshu Gupta, Bharathi Gorantla, Meena Gujrati, Dzung H. Dinh, Jasti S. Rao

Int J Oncol 36: 809-816, 2010; DOI: 10.3892/ijo_00000557

Following the publication of the above paper, it was drawn to the Editor's attention by a concerned reader that, for the immunocytochemical and immunohistochemical experiments shown in Fig. 5A-C on p. 815, several of the data panels exhibited overlapping sections, such that the data shown in the affected panels had all apparently been derived from the same original sources.

Owing to the large number of duplications of data that were identified in this paper, the Editor of *International Journal of Oncology* has decided that it should be retracted from the Journal on account of a lack of confidence in the presented data. The authors were asked for an explanation to account for these concerns, but the Editorial Office did not receive a reply. The Editor apologizes to the readership for any inconvenience caused.

